# An 18‐Month Randomized Controlled Clinical Trial Evaluating the Clinical Success of IPS e.max Conventional Crowns and Endocrowns in Extensively Restored Molars

**DOI:** 10.1002/cre2.70298

**Published:** 2026-01-26

**Authors:** Rand Essa Dalol, Jihad Nouman Abou Nassar, Mohammad Y. Hajeer

**Affiliations:** ^1^ Department of Fixed Prosthodontics, Faculty of Dentistry Syrian Private University Daraa Syria; ^2^ Department of Fixed Prosthodontics, Faculty of Dentistry University of Damascus Damascus Syria; ^3^ Department of Orthodontics, Faculty of Dentistry University of Damascus Damascus Syria; ^4^ Department of Orthodontics, School of Dentistry University of Jordan Amman Jordan

**Keywords:** clinical success, conventional crown, damaged molar teeth, endocrown, endodontically treated teeth, IPS e.max press crown, prosthodontic restorations

## Abstract

**Objectives:**

This RCT study aimed to investigate the clinical performance of IPS e.max endocrowns as an alternative option compared to conventional crowns to restore damaged molar teeth after an 18‐month follow‐up.

**Materials and Methods:**

A sample of 30 patients with 40 molars, who needed a single‐tooth restoration, was enrolled to receive either a conventional crown (*n* = 20) or an endocrown (*n* = 20). After molar preparation, all crowns were manufactured with the IPS e.max press system, then cemented using dual‐cure resin. All crowns were assessed using the modified United States Public Health Service criteria (USPHS) at baseline, 6, 12, and 18 months following placement. Patient satisfaction was evaluated using a questionnaire. Statistical analyses were performed using Mann‐Whitney U and Friedman tests (95% confidence interval).

**Results:**

All teeth in the conventional crown group and endocrown group showed 100% clinical success with no failure at 6, 12, and 18 months after placement in terms of marginal adaptation, contact points, and surface texture. No significant difference was found between endocrowns and conventional crowns in adhesive failure at 6 months (*p* = 0.075), 12 months (*p* = 0.317), and 18 months (*p* = 1.000). 100% of patients were very satisfied with the esthetics and comfort of the prosthesis. The function percentage was 93.3%.

**Conclusions:**

Both restorative options are durable and maintain their integrity. Adhesive failure occurred in a notable number of cases in the endocrown group compared to the conventional crown group. Patient satisfaction was high with both restoration types.

## 1 Introduction

The clinical success of endodontically treated teeth depends on many factors, one of which is the post‐endodontic restoration. When the remaining dental tissues do not provide sufficient retention for conventional restorations, treatment may be carried out using a post and core technique (Asmussen et al. 2005; Sirimai et al. 1999). Root canal posts are designed for the retention of the crown restoration but not for root stabilization (Sorensen and Engelman 1990). Clinical experience has revealed certain problems with metal posts, including tooth fracture or debonding (Bergman et al. 1989). Moreover, there has been clinical evidence of more frequent root fractures when pre‐fabricated posts were used compared to other types of prosthodontic reconstruction of pulpless teeth (Testori et al. 1993; Asmussen et al. 1999).

With the development of adhesive dentistry, the need for using posts and filling cores has decreased. Moreover, the appearance of ceramics with high mechanical strength and the capability of being acid‐etched, in addition to the adhesive capacity of resin cements, has allowed the restoration of posterior teeth without cores and intraradicular posts (Biacchi and Basting 2012). It is now feasible to restore posterior teeth with extensive loss of tooth structure by means of onlay and/or overlay restorations and, more recently, with the endocrown, without the use of posts and using the entire extension of the pulp chamber as a retentive resource (Zarow et al. 2009; Leirskar et al. 2003).

The ceramic monoblock technique for broken‐down teeth was described for the first time by Pissis (Pissis 1995) in 1995, and then Bindl and Mörmann (Bindl and Mörmann 1999) named this restorative procedure “endocrown” in 1999. The endocrown is a total porcelain crown fixed to a pulpless posterior tooth, which is anchored to the internal portion of the pulp chamber and to the cavity margins, thus obtaining macromechanical retention provided by the pulpal walls and microretention by using adhesive cementation (Lander and Dietschi 2008; Veselinovic et al. 2008).

The advent of ceramic materials, such as CAD‐CAM (computer‐aided design and manufacturing) and press systems, revitalized the possibility to produce single‐unit restorations characterized by high biocompatibility and good mechanical properties (Bindl et al. 2005).

Some laboratory and clinical studies have been made to evaluate the effectiveness (Lander and Dietschi 2008; Otto 2004), feasibility (Biacchi and Basting 2012; Bindl and Mörmann 1999; Göhring and Peters 2003), and clinical performance (Lander and Dietschi 2008; Veselinovic et al. 2008; Bernhart et al. 2010; Chaio et al. 2009) of endocrowns as a restorative procedure for endodontically treated teeth, in addition to the systematic reviews and prospective and retrospective studies (Leitão et al. 2022; Mario et al. 2022; Dewan 2023) about endocrown survival.

A critical analysis of the literature reveals significant inconsistencies regarding the primary failure modes and success rates of endocrowns, which appear to be influenced by several key factors. While a majority of studies reported debonding as the predominant failure mechanism (Lander and Dietschi 2008; Veselinovic et al. 2008; Chaio et al. 2009; Bernhart et al. 2010), others cited fracture (Govare and Contrepois 2020), or color mismatch (Otto 2004). This divergence can be critically examined through the lens of material selection, preparation design, and bonding protocols.

The high prevalence of debonding reported by multiple authors (Lander and Dietschi 2008; Veselinovic et al. 2008; Chaio et al. 2009; Bernhart et al. 2010) strongly suggests that the bonding interface is a critical vulnerability. However, the root cause of this debonding is likely multifactorial. For instance, the choice of material (e.g., glass‐ceramics vs. zirconia) directly influences bond strength and stress distribution. Furthermore, preparation geometry is a major source of conflicting evidence. While Mostafavi et al. (2022) recommend simpler cavity configurations to avoid non‐repairable fractures, other studies achieving high success rates may have utilized more retentive forms. This inconsistency highlights a lack of standardization in preparation design.

The reported survival rates themselves are inconsistent. Al‐Dabbagh (2021), in his SR, included three clinical and seven in‐vitro studies, reported a notably lower success rate (77.7%) for endocrowns compared to conventional crowns (94%). In contrast, more recent SR, like Leitão et al. (2022)—included nine clinical studies—reported (100%) success rate for endocrowns after a 3‐year follow‐up. This discrepancy may be attributed to variations in occlusal load distribution in the studied populations, or to the aforementioned factors of material, geometry, and follow‐up period, potentially reflecting improved bonding techniques or material advancements. Ultimately, the literature does not present a unified picture of failure; rather, it demonstrates that failure mode and rate are highly dependent on specific clinical and technical choices, explaining the divergent conclusions across studies.

Thus, additional well‐designed clinical trials are always required to strengthen the evidence regarding the use of endocrowns. The clinical relevance of this study is to determine the best restoration strategy for endodontically treated molars with extensive coronal destruction but a well‐preserved pulp chamber floor. In this common scenario, this study directly compared the clinical performance of two IPS e.max Press restoration designs: an endocrown versus a conventional crown retained on a composite core extended into the pulp chamber, after an 18‐month follow‐up period. The 18‐month follow‐up period was selected as it aligns with the timeframe used in previous studies (Ribeiro et al. 2024; Do et al. 2024; Moaleem and Al Ahmari 2024), allowing for direct comparison of our results with the existing literature. This study was designed to assess the medium‐term performance of these restorations where technical complications such as debonding, marginal discoloration, and early fractures in adhesive endocrowns and ceramic crowns manifest within the first 12–24 months of service, before long‐term wear and fatigue mechanisms become the dominant factors.

The null hypothesis (H₀) was that there was no significant difference in the clinical performance between the two groups. The alternative hypothesis (H₁) was that a significant difference existed between the two restoration types.

## 2 Materials and Methods

### 2.1 Study Design and Settings

This randomized controlled trial was conducted at the Department of Fixed Prosthodontics, Faculty of Dentistry, Damascus. This trial protocol was registered in the Clinical Trials Database (NCT02110550) and was partially funded by the University of Damascus (reference number: 501100020595).

### 2.2 Sample Size Estimation

The sample size was determined using G*Power 3.1.3 at an alpha level of 0.05 and a 95% confidence interval. The calculated sample size was 38 participants (19 per group). An additional two patients were added, one patient to each group, to account for potential dropouts.

### 2.3 Patient Recruitment and Inclusion Criteria

After examining 54 patients attending the Department of Fixed Prosthodontics, Faculty of Dentistry, University of Damascus, who suffered from broken‐down molar teeth, 40 patients (aged 22–50) met the requirements for inclusion. Patients were informed about the treatment options of a conventional crown or an endocrown, for which long‐term results are still lacking, using standardized and well‐detailed information sheets. Upon accepting to participate in the trial, informed consent forms were obtained from patients. Of the 40 patients who consented to participate in the trial, 30 (13 men and 17 women) were chosen randomly. All the participants met the following inclusion criteria: (1) adult patients aged 20–60; (2) first and second upper and lower damaged molars only; (3) at least two remaining axial walls; (4) at least zero point 5 millimeters above the gingival line for damaged axial walls; (5) good endodontic treatment and apical seal; and (6) opposing teeth could be natural or crowned. The criteria for exclusion were: (1) patients with low oral hygiene; (2) gingival inflammation; (3) parafunctional habits; (4) users of removable partial dentures; (5) systemic or local drug use that could provide an adverse effect on gingival health; and (6) systemic illnesses or conditions that affect periodontal tissues.

### 2.4 Randomization and Allocation Concealment

Simple randomization was carried out to assign the 40 molars into two groups: (1) conventional crown, and (2) endocrown. An academic member who was not involved in this research created a list of random numbers using Minitab® Version 17.1 (Minitab Inc., State College, PA, USA), with an allocation sequence of a 1:1 ratio. Each number was written on white paper and placed into a plain opaque envelope. The randomization was for crown selection (conventional crown or endocrown); the sequentially numbered, sealed, and opaque envelopes containing the allocation sequence were then opened. Blinding was applied only to the outcomes’ assessor, and it did not apply to either the patient or the principal investigator.

### 2.5 Teeth Preparation, Impression, Construction, and Cementation

#### 2.5.1 The First Experimental Group: The Conventional Crown Group

**Figure 1:**
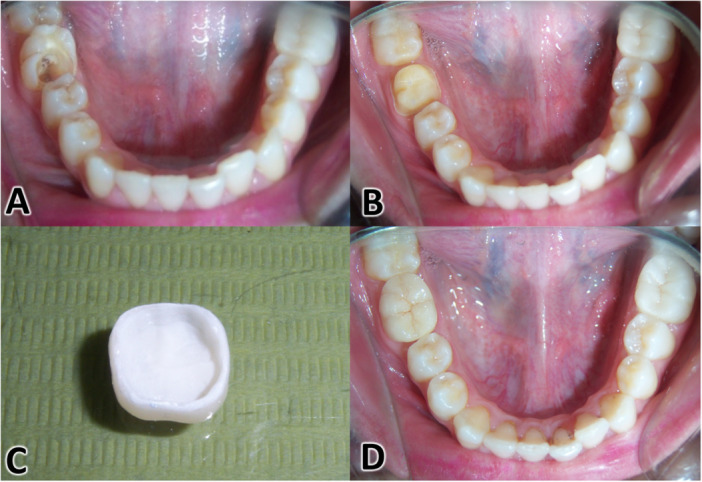
Clinical aspects of a conventional crown from preparation to placement. (A) An endodontically treated right mandibular first molar; (B) conventional crown preparation; (C) The conventional crown after conclusion of the work in the prosthesis laboratory; (D) Cementation of the conventional crown.

The steps of molar preparation are shown in Figure 1. Damaged molar teeth were prepared to receive a composite resin restoration as follows: 1‐ the pulp chamber and internal sides of the remaining axial walls were prepared to remove undercuts and ensure a central retention cavity using a round end tapered bur #850.010 C (Nti Diamant instruments); then, a composite resin core (ScotchBond Multi‐Purpose and Z250, 3 M ESPE) was built up starting from the pulp chamber to the occlusal surface of the molar.

The restored molar was prepared to receive a lithium disilicate conventional crown (IPS e.max Press, Ivoclar Vivadent, Liechtenstein) as follows: (1) two millimeters were cut from the occlusal surface using a fissure bur #835.010 SC (Nti Diamant instruments, Germany); (2) 1.5 mm were cut from the axial walls using a round end tapered bur #850.010 C (Nti Diamant instruments, Germany), resulting in a 0.8‐mm wide circumferential deep chamfer at the gingival margins. The preparation was finished, and the axial walls were polished using a smooth diamond cylinder bur #836kr.314.014 (Komet Dental, Gebr. Brasseler, Lemgo, Germany).

Retraction cords were placed (Ultrapake #00, Ultradent, USA) and impressions were made with condensation silicone (putty/light body, Zetaplus, Oranwash, Zhermack, Italy) using a two‐step technique. The interocclusal relation was recorded in centric occlusion position using bite wax (Tenatex Red, Kemdent, UK). The indirect temporary crowns, made of self‐cure acrylic resin (Structure 2 SC, Voco, Cuxhaven, Germany) in the dental lab based on the primary alginate impression of each molar, were bonded using temporary eugenol‐free cement (Vision Provisory, Base, Catalyst, Turkey).

Impressions were sent to the dental lab to fabricate the crowns from IPS e.max Press ingots (IPS e.max Press, Ivoclar Vivadent, Liechtenstein) using the lost‐wax technique, after selecting the suitable shade for each patient. All molars were prepared by the principal investigator (RD) and fabricated in her practice‐based laboratory to ensure strict standardization of the restoration quality and fit, which could have introduced variability if outsourced to multiple technicians. To mitigate the potential bias and ensure the objectivity of the clinical assessments, all post‐cementation evaluations were performed by an independent, calibrated examiner who was not involved in the treatment phase and was blinded to the restoration type.

#### 2.5.2 The Second Experimental Group: The Endocrown Group

**Figure 2:**
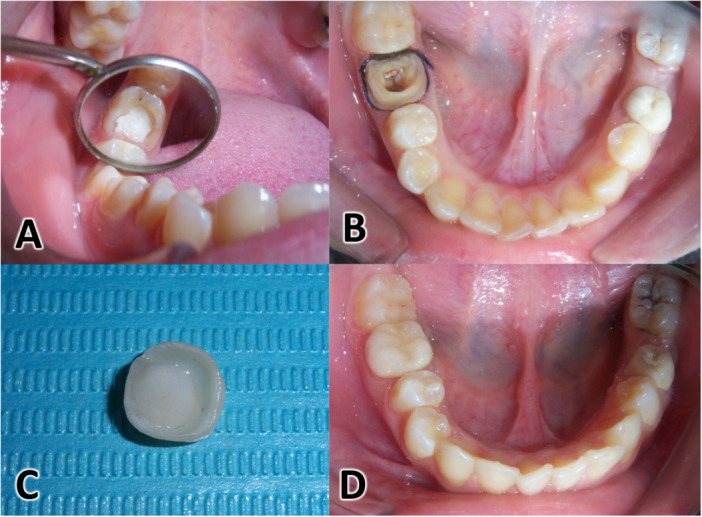
Clinical aspects of an endocrown from preparation to placement. (A) An endodontically treated right mandibular first molar; (B) endocrown preparation; (C) The endocrown after conclusion of the work in the prosthesis laboratory; (D) Cementation of the endocrown.

The steps of molar preparation are shown in Figure 2. All broken‐down molar teeth were prepared to receive a lithium disilicate endocrown (IPS e.max Press, Ivoclar Vivadent, Liechtenstein) as follows: (1) 2 mm were cut from the occlusal surface using a fissure bur #835.010 SC (Nti Diamant instruments, Germany); (2) the pulp chamber and internal sides of the remaining axial walls were prepared to confirm 6–10 degrees of open access, with a central retention cavity and rounded internal line angles using a round end tapered bur #850.010 C (Nti Diamant instruments); (3) the same bur was used to cut 1.5 mm from the external sides of the axial walls, resulting in a 0.8‐mm wide circumferential deep chamfer at the gingival margins. The preparation was finished, and the axial walls were smoothed using a smooth diamond cylinder bur #836kr.314.014 (Komet Dental, Gebr. Brasseler, Lemgo, Germany). The upcoming steps were similar to the conventional crown group: retraction cords were placed (Ultrapake #00, Ultradent, USA) and impressions were made with condensation silicone (putty/light body, Zetaplus, Oranwash, Zhermack, Italy) using a two‐step technique. The interocclusal relation was recorded in centric occlusion position using bite wax (Tenatex Red, Kemdent, UK). The indirect temporary crowns, made of self‐cure acrylic resin (Structure 2 SC, Voco, Cuxhaven, Germany) in the dental lab based on the primary alginate impression of each molar, were bonded using temporary eugenol‐free cement (Vision Provisory, Base, Catalyst, Turkey). Impressions were sent to the dental lab to fabricate the endocrowns from IPS e.max Press ingots (IPS e.max Press, Ivoclar Vivadent, Liechtenstein) using the lost‐wax technique, after selecting the suitable shade for each patient. All molars were prepared by the principal investigator (RD) and fabricated in her practice‐based laboratory to ensure strict standardization of the restoration quality and fit.

### 2.6 Cementation for Both Types of Crowns

The finished crowns and endocrowns were checked for shade, fit, and occlusion in the patient's mouth and then bonded using dual‐cure resin cement (Variolink, Ivoclar Vivadent, Schaan/Liechtenstein). Isolation was achieved using a high‐volume suction, cotton rolls in the vestibule and floor of the mouth, and dry‐angles placed over the parotid ducts. The internal surface of the crowns and endocrowns were treated in accordance with the technique recommended for lithium disilicate‐based ceramics: (1) application of 5% hydrofluoric acid (Porcelain Etching Gel, Ultradent, USA) on the internal surface for 20 s; (2) rinsed with water/air for 30 s; (3) application of the silane agent (Ultradent Silane, USA) for 1 min; (4) the molar was etched with 35% phosphoric acid (CharmEtch, DentKist, Korea) for 15 s; afterward, it was rinsed with abundant water, and an air jet was applied for 20 s; the preparation was dried, keeping the dentin moist; (5) a thin coat of the adhesive agent (Tetric N‐Bond, Ivoclar Vivadent, USA) was applied using a disposable applicator (TPC Disposable Micro Applicator, CA), followed by a light air jet and light activation for 10 s; and (6) the dual‐cure resin cement (Variolink, Ivoclar Vivadent, USA) was applied on the crowns and endocrowns, which were then placed in position on the preparation. The excess cement was removed using a disposable applicator, and then light activation was applied for 40 s on the occlusal, buccal, and lingual surfaces.

### 2.7 Outcome Measures

All crowns were assessed using the modified US Public Health Service (USPHS) criteria (Cvar and Ryge 2005; Sun et al. 2019; Hickel et al. 2010), where marginal adaptation, contact points, surface texture, color match, and adhesive failure were included. Every parameter was rated as follows: Alpha: indicates an excellent clinical performance with no detectable issues; Bravo: signifies a satisfactory clinical performance with minor issues that do not significantly compromise the restoration's function or longevity; Charlie: this rating reflects a restoration with noticeable deficiencies; and Delta: indicates treatment failure, with significant issues necessitating immediate intervention or replacement of the restoration.

All crowns in both groups were rated at baseline and after 6, 12, and 18 months. The clinical evaluation was conducted using a mouth mirror and an explorer (EXD56, Hu‐Friedy, USA), waxed dental floss (Essentialfloss, Oral B, Ireland), radiographs to assess the mesial and distal marginal adaptation, and a color guide (Vita Lumin vacuum shade guide, Germany) which is a qualitative rating method that assesses the clinical acceptability of the color match between the restoration and the adjacent tooth structure to evaluate whether the restoration is esthetically acceptable in its clinical context.

Patient satisfaction was assessed using a questionnaire. Few steps were taken to ensure its validity and reliability. The questionnaire items were developed based on a comprehensive review of the literature (Otto and Mörmann 2015; Zou et al. 2018; Belleflamme et al. 2017) and were reviewed by a panel of two prosthodontists and a biostatistician to ensure they comprehensively covered all relevant domains of patient satisfaction (e.g., esthetics, function, comfort). Then it was piloted on a small group of patients who were not included in the main study to ensure the questions were clear, unambiguous, and easy to understand.

### 2.8 Statistical Analysis

All statistical analyses were performed using SPSS Statistics for Windows, Version 13.00 (SPSS Inc, Chicago, IL, USA). A significance level of 0.05 and a 95% confidence interval were used to evaluate the results. The median (lowest‐maximum) and N (%) were used to express descriptive statistics. The Mann‐Whitney U and Friedman tests were used to identify statistically significant changes between both groups over the study period.

## 3 Results

### 3.1 Baseline Sample Characteristics

**Figure 3:**
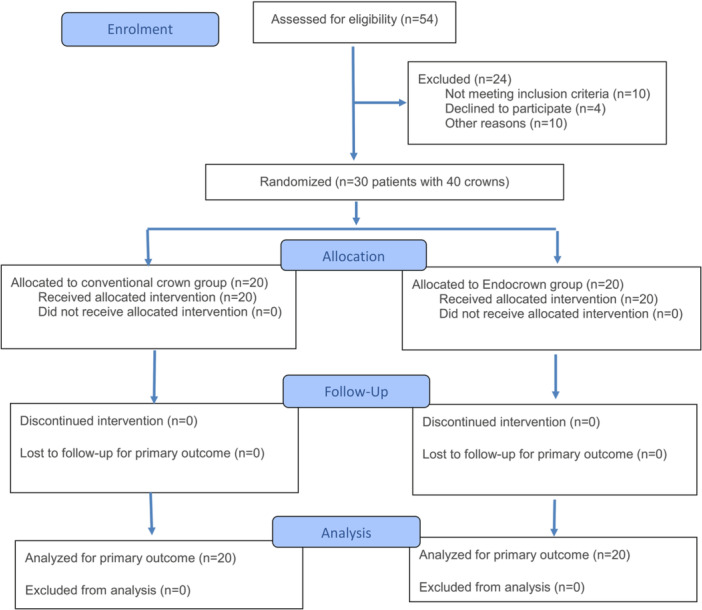
The Consolidated Standards of Reporting Trials (CONSORT) flow diagram of patient recruitment, assignment, follow‐up, and inclusion in data analysis.

The Consolidated Standards of Reporting Trials (CONSORT) flow diagram illustrates patient recruitment, assignment, follow‐up, and inclusion in data analysis (Figure 3). In total, 30 patients with 40 crowns were enrolled (13 males (43.3%) and 17 females (56.7%)) in the study groups, with a mean age of 27.7 ( ± 8.1) years (Table 1). A total of 20 crowns were enrolled in the conventional crown group, and 20 endocrowns were enrolled in the endocrown group. There were no dropouts.

**Table 1:** Baseline sample characteristics (age and gender).

Variable	Study sample (*n* = 30 Patients with 40 crowns)	
Age (In years)	Mean ± SD	27.7 ± 8.1
Gender	Male	13 (43.3%)
	Female	17 (56.7%)

### 3.2 Main Findings

Table 2 shows the modified USPHS rating of conventional crowns and endocrowns at baseline, 6 months, 12 months, and 18 months, including marginal adaptation, contact points, surface texture, color match, and adhesive failure. 100% of treated teeth in the conventional crown group and endocrown group achieved the Alpha grade 6, 12, and 18 months after placement in terms of marginal adaptation, contact points, and surface texture. However, at the 18‐month recall, a slight change in color match was observed. The Alpha rating decreased from 100% to 95% in both groups (five out of 20 crowns in each group scored Bravo). For the adhesive failure, the conventional crown group demonstrated high retention, with only a single failure where the crown lost retention and required replacement after 18 months, resulting in a 95% Alpha rating. Otherwise, the endocrown group showed a more dynamic pattern. Three restorations (15%) had Charlie ratings (partially debonded but retainable) at the 6‐month recall. This improved by 12 months, with only one Charlie rating, suggesting stabilization. However, at 18 months, one restoration (5%) experienced a complete loss of retention (Delta rating), requiring replacement.

**Table 2:** Modified United States Public Health Service rating of the conventional crown and endocrown groups at baseline, 6 months, 12 months, and 18 months after treatment, n (%).

Quality assessment	Conventional crown group					Endocrown group					
	Alpha	Bravo	Charlie	Delta	P a	Alpha	Bravo	Charlie	Delta	P^a^	P^b^
**Adhesive Failure**											
Baseline	20 (100)	0	0	0		20 (100)	0	0	0		1.000
6 months	20 (100)	0	0	0		17 (85.0)	0	3 (15.0)	0		0.075
12 months	20 (100)	0	0	0	0.392	19 (95.0)	0	1(5.0)	0	0.284	0.317
18 months	19 (95.0)	0	0	1 (5.0)		19 (95.0)	0	0	1(5.0)		1.000
**Color match**											
Baseline	20 (100)	0	0	0		20 (100)	0	0	0		1.000
6 months	20 (100)	0	0	0	0.002	20 (100)	0	0	0	0.002	1.000
12 months	20 (100)	0	0	0		20 (100)	0	0	0		1.000
18 months	15 (95.0)	5(25.0)	0	0		15 (95.0)	5 (25.0)	0			1.000
**Marginal adaptation**											
Baseline	20 (100)	0	0	0	N.A	20 (100)	0	0	0	N.A	
6 months	20 (100)	0	0	0		20 (100)	0	0	0		
12 months	20 (100)	0	0	0		20 (100)	0	0	0		
18 months	20 (100)	0	0	0		20 (100)	0	0	0		
Baseline	20 (100)	0	0	0	N.A	20 (100)	0	0	0	N.A	
6 months	20 (100)	0	0	0		20 (100)	0	0	0		
12 months	20 (100)	0	0	0		20 (100)	0	0	0		
18 months	20 (100)	0	0	0		20 (100)	0	0	0		
**Surface texture**											
Baseline	20 (100)	0	0	0	N.A	20 (100)	0	0	0	N.A	
6 months	20 (100)	0	0	0		20 (100)	0	0	0		
12 months	20 (100)	0	0	0		20 (100)	0	0	00		
18 months	20 (100)	0	0	0		20 (100)	0	0	0		

*Note:* N.A: not applicable since the values are constant along the evaluated time points.

^a^ Friedman test.

^b^ Mann‐Whitney test.

The Mann‐Whitney U test revealed no significant difference between endocrowns and conventional crowns in adhesive failure at 6 months (*p* = 0.075), 12 months (*p* = 0.317), and 18 months (*p* = 1.000). The Friedman test revealed no significant difference between the endocrown group (*p* = 0.284) and the conventional crown group (*p* = 0.392) in adhesive failure. No caries or endodontic problems were observed after the 18‐month follow‐up examination.

Patient satisfaction is presented in Table 3. One hundred percent of patients were very satisfied with the esthetics and comfort of the prosthesis. The function percentage was 93.3%. No one expressed a neutral or unsatisfied attitude toward the treatment. In general, 100% were delighted with the endocrown and conventional crown.

**Table 3:** Patient satisfaction following treatment, *n* (%).

Item	Very satisfied	Satisfied	Neutral	Unsatisfied	Very unsatisfied
Esthetic	30 (100)	0	0	0	0
Function	28 (93.3)	2 (6.6)	0	0	0
Uncomfortable feelings	30 (100)	0	0	0	0
General evaluation	30 (100)	0	0	0	0

## 4 Discussion

This study was performed to provide more information about the endocrown as a technique for crowning broken‐down molar teeth compared to one of the traditional ways of treating these cases.

Forty molars from 30 patients were treated in this study. All patients were between 19 and 45 years old. The percentages of men and women were 43.3% and 56.7%, respectively. 73.3% of patients had one molar that needed to be crowned; 20.0% had two molars; and 6.7% had three molars. 75.0% of molars in this study were first molars, and 25.0% were second molars. 27.5% of molars were maxillary, and 72.5% were mandibular.

According to Zou et al. (2018), the endocrown is mainly indicated for molars. The molars were selected because there is a greater surface area accessible for bonding on molars compared to premolars, where the smaller tooth structure in the pulp chamber reduces the bonding surface, limiting the bonding strength of the adhesive system and the resin (Bindl et al. 2005).

The crown base‐to‐crown height ratio may cause larger leverage for premolars compared to molars, increasing the risk of restorative fracture (Veselinovic et al. 2008). A systematic review reported that the survival rate is higher for molars than for premolars (Papia et al. 2020). Hence, only molars were selected for the study.

In this study, the endocrown preparation was conducted according to previous studies (Fages and Bennasar 2013; Sevimli et al. 2015; Menezes‐ Silva et al. 2016). Impressions were performed using condensation silicone with a two‐step technique, similar to previous studies (Veselinovic et al. 2008; Menezes‐ Silva et al. 2016).

Lithium disilicate (IPS e.max Press, Ivoclar Vivadent, Liechtenstein) was used in this study because it is one of the recommended ceramic types for manufacturing the endocrown (Pissis 1995), and it was reported that the adhesive qualities of lithium disilicate ceramics make them one of the best materials for restoration (Gresnigt et al. 2016; Godil et al. 2021). The pressable ceramic system with the lost‐wax technique was used in this study because it was reported to copy the pulp chamber details better than the CAD‐CAM technique (Veselinovic et al. 2008; Guess et al. 2014).

Dual‐cure resin cement (Variolink, Ivoclar Vivadent, Schaan/Liechtenstein) was used for cementation in this study, according to previous studies (Papia et al. 2020; Menezes‐ Silva et al. 2016; Thomas et al. 2020).

### 4.1 Marginal Adaptation

In this study, both conventional crowns and endocrowns were recorded as Alpha grade regarding marginal adaptation at baseline, 6 months, 12 months, and 18 months follow‐up. There was no gap between the tooth tissue and the restoration using a sharp probe. The marginal integrity observed in both restoration types throughout the study period can be critically synthesized as a function of material properties and adhesive performance, rather than restoration design alone. The consistent “Alpha” ratings for both conventional crowns and endocrowns suggest that when a high‐strength, well‐adapted lithium disilicate (IPS e.max) restoration is luted with a meticulous adhesive protocol, the resulting marginal seal is highly resistant to degradation in the short‐to‐medium term.

The concordance of our results with studies utilizing pressed ceramics (Etman and Woolford 2010; Esquivel‐Upshaw et al. 2013) and the divergence from studies reporting higher degradation with CAD/CAM systems (Otto and Schneider 2008) support a hypothesis that fabrication method may be a more significant predictor of marginal adaptation than the macro‐design of the crown. As suggested by Guess et al. (2014), the superior internal fit of the press technique likely creates a more favorable and uniform resin cement layer, minimizing localized stress and microleakage at the margins. Therefore, the clinical decision between an endocrown or conventional crown depends on the selected fabrication method to ensure it achieves an optimal fit, in addition to other factors like pulp chamber depth and operative steps. Mostafavi et al. 2022), and quality and quantity of the remaining dentine substrate (Yu and Wang 2014; Kwong 2002; Karakaya et al. 2008).

### 4.2 Contact Points

The consistent ‘Alpha’ rating for contact points in both groups over the 18‐month period indicates a notable stability in the proximal contours of lithium disilicate restorations. The absence of any change suggests that the high wear resistance and structural integrity of pressed IPS e.max ceramic are sufficient to maintain anatomical form and proximal relationships, regardless of the restoration's macro‐design. A previous study corresponding with our results revealed the priority of IPS e.max Press crowns over other ceramic systems due to the absence of wear facets and surface roughness after a 3‐year follow‐up (Etman and Woolford 2010). Similar results were reported in Otto's study (2004), where no change was recorded in either the anatomic form or surface texture of Cerec conventional crowns and endocrowns after a 12‐month follow‐up. Our results revealed that the material and its fabrication process are the main factors in clinical performance, in addition to restoration type.

### 4.3 Surface Texture

The maintenance of an “Alpha” grade for surface texture across all restorations underscores the durability of modern high‐strength ceramics in the short‐to‐medium term. The absence of roughness, cracks, or fractures at 18 months indicates that IPS e.max lithium disilicate possesses sufficient flexural strength to withstand masticatory forces without surface degradation, provided an optimal adhesive bond is present. The absence of fractures in our cohort aligns with previous studies (Veselinovic et al. 2008; Otto 2004; Carlos et al. 2013). A previous systematic review (Sedrez‐Porto et al. 2016) included three clinical endocrown studies that reported no change in surface texture with high success rates (94%–100%) up to a 36‐month follow‐up. On the other hand, our results contrast with earlier studies that reported failures. This divergence can be critically synthesized as evidence of material superiority. The fractures reported in studies using Empress II (Toksavul and Toman 2007)—a material with a known lower flexural strength—and in earlier CAD/CAM systems (Bindl and Mörmann 1999) highlight a key insight that clinical success in terms of fracture resistance is less about the endocrown concept itself and more about the material properties of the ceramic used to execute it. Therefore, the 1.5% fracture rate reported by Papia et al. (2020) represents a historical average diluted by earlier materials, while our results point to the lithium disilicate.

### 4.4 Color Match

Color match was assessed visually using a color guide (Vita Lumin vacuum shade guide, Germany), similarly to previous studies (Otto 2004; Cvar and Ryge 2005). Color mismatch may be caused by several factors, such as alterations in dual‐cure resin cement under the crowns (Walls et al. 2002), wearing out of the external staining layer of the crowns (Donovan 2008), change in color and clarity of natural teeth in the oral environment (Zou et al. 2018), in addition to other individual reasons regarding smoking, diet habits, and oral hygiene.

The emergence of a slight color mismatch (Bravo grade) in 25% of restorations in both groups at the 18‐month mark reveals a time‐dependent limitation not captured by shorter‐term studies. The fact that this change occurred with equal frequency in both conventional crowns and endocrowns critically shifts the focus away from restoration design and toward a common underlying mechanism. This pattern strongly implicates the aging of the resin cement layer and/or the ceramic surface as the primary culprits, rather than an intrinsic flaw in either crown type. Similar results to the current study were reported in a previous research that recorded a clinically acceptable A or B rating regarding the color match of 20 Cerec crowns and endocrowns after a 16‐month follow‐up (Otto 2004). Taskonak and Sertgöz (2006) reported in their study a slight color mismatch of IPS e.max Press crowns after a 24‐month follow‐up.

Our 18‐month results stand in contrast to the stable color reported by Veselinovic et al. (2008) at 12 months and Carlos et al. (2013) at 24 months. This discrepancy may be attributable to differences in the ceramic systems (CAD/CAM vs. pressed) and the resin cements used, whose color stability can vary significantly between products. This divergence suggests that the 12–18‐month period may be a critical window for the onset of visually detectable changes in the adhesive‐ceramic‐tooth complex. The color stability maintained for the first 12 months demonstrates the high initial color fidelity of the IPS e.max material. However, the slight color mismatch (Bravo grade) observed in 25% of our restorations at 18 months may be attributed to a combination of subsurface and surface factors. While the monolithic IPS e.max Press ceramic itself has demonstrated excellent color stability in vitro, its external characterization stains are susceptible to degradation over time. This phenomenon is replicated in artificial aging studies, where thermocycling and exposure to staining solutions like coffee and red wine can lead to measurable color changes by altering the stained surface (Sedanur and Bora 2011). This is supported by the findings of Meral and Bilge (2019), who showed that artificial aging can cause significant color change in externally characterized monolithic ceramics, confirming that this surface treatment is susceptible to change over time. (Meral and Bilge 2019).

The subsequent change underscores that the long‐term esthetic outcome is not solely dependent on the ceramic itself but is a function of the entire system. Therefore, future efforts to enhance the longevity of esthetic restorations should prioritize the development of more color‐stable resin cements and the optimization of surface treatments to protect the glaze layer.

### 4.5 Adhesive Failure

This study reported 3/20 endocrowns recorded as Charlie grade (partially debonded but retainable) in adhesive failure after 6 months; 1/20 Charlie grade after 12 months, and 1/20 Delta grade (complete loss of retention) after 18 months because it lost retention and needed to be replaced. Many previous studies (Lander and Dietschi 2008; Veselinovic et al. 2008; Chaio et al. 2009; Bernhart et al. 2010) reported debonding as the main disadvantage of endocrowns. Mario et al. (2022), in a systematic review that included four clinical ceramic endocrown studies, reported adhesive failure of 16 endocrowns after a 7–19 year follow‐up. The repeated adhesive failure was justified by Zarone et al. (2006), who studied several types of fixed restorations from different materials on maxillary incisors. Their 3D finite element analysis showed that critical areas of high stress in conventional restorations on endodontically treated teeth were located in the restoration‐cement‐dentine zone, which led to loss of retention. In this study, the primary contributing factor identified for debonding was the quality and quantity of the remaining dentine substrate. This conclusion is based on the Location of Failure, in which the adhesive failure was observed at the dentin‐cement interface, indicating a challenge in achieving adequate adhesion to the available substrate. Multiple studies (Yu and Wang 2014; Kwong 2002; Karakaya et al. 2008) have consistently demonstrated that bond strengths to sclerotic dentin are significantly lower than to normal dentin, with morphological analyses revealing thinner hybrid layers and shorter resin tags. Also, it was reported that bond strengths to pulp chamber floor dentin are significantly lower than to other regions of the tooth according to several studies (Akagawa et al. 2002; Kaptan et al. 2019).

This study showed that one of the disadvantages of the endocrown was the sensitivity in the impression and cementation steps, where isolation was challenging, and that may be considered one of the factors that led to the debonding. While acknowledging the gold‐standard status of the rubber dam, this study was designed to reflect a realistic clinical environment in a specialist prosthodontic practice or university hospital setting or else. In this study, isolation was achieved for all cementation procedures using a combination of high‐volume suction, cotton rolls in the vestibule and floor of the mouth, and dry‐angles placed over the parotid ducts. The results of the current study emphasize applying the rubber dam to ensure perfect isolation during the adhesive procedure.

This study eliminated pulp chamber depth through standardized preparation. Also, it eliminated occlusal forces from debonding reasons because all restorations were checked and adjusted at the cementation appointment to ensure even occlusal contacts without premature contacts. The debonding did not appear to be associated with specific parafunctional habits in these patients. The clinical debonding findings in this study are mechanistically explained by the in‐vitro work of (Waleed 2023), whose finite element analysis demonstrated high stress concentrations at the adhesive interface of endocrowns, particularly under oblique loading.

### 4.6 Patient's Satisfaction

In this study, all patients were “very satisfied” with the esthetics (100%), most patients were “satisfied” (93.3%) with the function of the restoration, all patients (100%) felt comfortable with the crowns, and were “very satisfied” with the general evaluation. This high satisfaction occurred despite the objective measurement of color mismatch (Bravo grade) in 25% of restorations at 18 months, which demonstrates that the slight, time‐dependent color changes, while detectable by a clinician's critical eye, often fall within a patient's threshold of esthetic acceptance.

These rates are consistent with a small number of studies that have evaluated patient satisfaction with endocrown treatment. Otto and Mörmann (2015) only reported that all patients were “very satisfied” and “satisfied” with the treatment results, regardless of the criteria. This is similar to Belleflamme et al.‘s study (2017), where the “very satisfied” and “satisfied” rates were 92.9% and 2%, respectively. According to Zou et al.‘s study (2018), the assessment of patient satisfaction was based on three criteria: color, appearance, and comfort.

The endocrown technique was reported to have several advantages over conventional crowns, such as a reduced number of interfaces in the restorative system, less stress concentration because of the reduction in the non‐homogenous material present (Chaio et al. 2009; Bernhart et al. 2010), minimal involvement of the biological width (Dietschi et al. 2011), and in comparison to post and core restorations, the bonding surface offered by the pulpal chamber of the endocrown was found to be equal or even superior to that obtained from the bonding of a radicular post of 8 mm depth (Rocca and Serge 2008).

This study showed that the endocrown preparation design required more removal of remaining coronal tooth structure compared to conventional crowns, where more reduction was needed to remove any undercut inside the pulp chamber and to create wide open walls; after finishing the preparation, every remaining axial wall less than 1 mm in thickness had to be removed. This does not correspond with Dietschi et al. (2008), who found that endocrowns were more conservative than conventional crowns.

It was reported that endocrowns are indicated in cases where there are minimal functional and lateral stresses. When there is evidence of increased functional and lateral stresses, as evident with steep occlusal anatomy, wear facets, or parafunction, a full coverage crown with or without a post is the treatment of choice (Rocca and Serge 2008). However, the success and longevity of the endocrown are directly related to the correct preparation of the tooth, the selection of the most suitable ceramic options, and the choice of bonding material, since adequate adhesive cementation is absolutely necessary for the success of this restorative treatment (Bindl and Mörmann 1999; Bindl et al. 2005; Göhring and Peters 2003). A formal economic and time evaluation was beyond the scope of this study, but it represents a valuable direction for future research.

The findings from this 18‐month clinical study provide an evidence‐based framework for selecting between an endocrown and a conventional crown for an endodontically treated molar. This decision should be guided by the quantity and quality of the remaining coronal dentin and the operator's capacity to execute a perfectly controlled adhesive protocol. Conventional crowns are preferred over endocrowns under three primary conditions: (1) in severely compromised teeth with less than two axial walls, a minimal ferrule, and a shallow pulp chamber, where a core is necessary to create a retentive form; (2) in cases with anticipated challenges in isolation or moisture control, which elevate the risk associated with the endocrown's technique‐sensitive adhesive protocol; and (3) when the dentin substrate is suspected to be heavily sclerotic, thereby potentially compromising the adhesive bond. Therefore, the endocrown is not a universal replacement for the conventional crown but rather a superior option in well‐selected cases where its adhesive, tooth‐preserving principles can be fully realized.

### 4.7 Limitations

One of the limitations in this study is the small sample size, which was determined by an a priori power calculation and remains modest. This limits the statistical power of the analyses, increasing the risk of Type II errors, where true differences between the groups may not have been detected. Consequently, the findings should be interpreted as preliminary evidence, and their generalizability to broader populations may be limited. Future studies with a larger sample size are recommended to detect small effect sizes in which more significant differences could be reported as definitive evidence. Another limitation of the current study was the inability to blind the operators and patients due to the visible differences in the clinical procedures and materials used for each intervention. Additionally, having the operator fabricate the restorations presented a potential for bias. To mitigate these risks, all primary outcome measures were assessed by an independent, calibrated examiner who was blinded to the study groups. Also, the USPHS criteria are excellent for detecting overt clinical failures, but they may not capture the very earliest molecular signs of adhesive degradation. Future studies could be strengthened by using sensitive methods like micro‐CT or digital scanning to supplement the clinical assessments with quantitative data to detect sub‐clinical degradation. Furthermore, our clinical findings should be interpreted alongside future laboratory studies that investigate the underlying mechanical properties.

## 5 Conclusions

Based on our 18‐month findings, the clinical decision between an endocrown and a conventional crown for an endodontically treated molar extends beyond equivalent short‐term success. Endocrown's primary advantage may lie more in its procedural simplicity and adhesion‐based philosophy than in universally superior tooth structure preservation, as our results indicate that it required more removal of remaining coronal tooth structure than previously suggested. The higher rate of adhesive failure, even if manageable, signals that a critical point was not seen with conventional crowns. This suggests that case selection, meticulous adhesive technique, and patient factors are paramount for endocrown success. Ultimately, endocrowns long‐term predictability relative to conventional crowns remains to be fully established, underscoring the necessity for extended longitudinal studies.

## Author Contributions

Conceptualization, Rand Essa Dalol and Jihad Nouman Abou Nassar. methodology, Rand Essa Dalol and Jihad Nouman Abou Nassar. validation, Mohammad Y. Hajeer. investigation, Rand Essa Dalol. resources, Rand Essa Dalol. writing – original draft preparation, Rand Essa Dalol and Mohammad Y. Hajeer. writing – review and editing, Rand Essa Dalol and Mohammad Y. Hajeer. visualization, Rand Essa Dalol and Mohammad Y. Hajeer. supervision, Jihad Nouman Abou Nassar. All authors have read and agreed to the published version of the manuscript.

## Ethics Statement

Each participant provided written informed consent to participate in the study. The Local Research Ethics Committee of the University of Damascus approved the study (UDDS‐619‐21072013/SRC‐129).

## Conflicts of Interest

The authors declare no conflicts of interest.

## Data Availability

The datasets used and/or analyzed during the current study are available from the corresponding author upon reasonable request.
